# Overexpression of RAB27A in Oral Squamous Cell Carcinoma Promotes Tumor Migration and Invasion via Modulation of EGFR Membrane Stability

**DOI:** 10.3390/ijms241713103

**Published:** 2023-08-23

**Authors:** Jue Huang, Jie-Gang Yang, Jian-Gang Ren, Hou-Fu Xia, Gao-Hong Chen, Qiu-Yun Fu, Lin-Zhou Zhang, Hai-Ming Liu, Kui-Ming Wang, Qi-Hui Xie, Gang Chen

**Affiliations:** 1 State Key Laboratory of Oral & Maxillofacial Reconstruction and Regeneration, Key Laboratory of Oral Biomedicine Ministry of Education, Hubei Key Laboratory of Stomatology, School & Hospital of Stomatology, Wuhan University, Wuhan 430079, Chinaliuhaiming@whu.edu.cn (H.-M.L.);; 2 Department of Oral and Maxillofacial Surgery, School & Hospital of Stomatology, Wuhan University, Wuhan 430079, China

**Keywords:** oral squamous cell carcinoma, metastasis, RAB27A, EGFR, palmitoylation

## Abstract

Oral squamous cell carcinoma (OSCC) is the most prevalent subtype of head and neck tumors, highly prone to lymph node metastasis. This study aims to examine the expression pattern of Ras-related protein Rab-27A (RAB27A) and explore its potential implications in OSCC. The expression of RAB27A was assessed through immunohistochemical analysis utilizing tissue microarrays. In vitro experiments were conducted using RAB27A-knockdown cells to investigate its impact on OSCC tumor cells. Additionally, transcriptome sequencing was performed to elucidate potential underlying mechanisms. RAB27A was significantly overexpressed in OSCC, and particularly in metastatic lymph nodes. It was positively correlated with the clinical progression and poor survival prognosis. Silencing RAB27A notably decreased the proliferation, migration, and invasion abilities of OSCC cells in vitro. A Gene Ontology (GO) enrichment analysis indicated a strong association between RAB27A and the epidermal growth factor receptor (EGFR) signaling pathway. Further investigations revealed that RAB27A regulated the palmitoylation of EGFR via zinc finger DHHC-type containing 13 (ZDHHC13). These findings provide insights into OSCC progression and highlight RAB27A as a potential therapeutic target for combating this aggressive cancer.

## 1. Introduction

Oral squamous cell carcinoma (OSCC) is the most common subsite of head and neck tumors, with an overall five-year survival rate of less than 60% due to its aggressive behavior and propensity for lymph node metastasis [1]. In the past few decades, despite numerous studies attempting to elucidate the mechanisms of progression of OSCC [2,3,4], current treatment strategies do not successfully address the issues of invasion and metastasis in OSCC. As a result, a significant number of patients still experience rapid recurrence [5].

The epidermal growth factor receptor (EGFR) signaling pathway has emerged as a pivotal player among the elucidated mechanisms, significantly influencing the onset and progression of OSCC [6], as up to 80–90% of patients overexpress or harbor mutations in the EGFR, directly impacting overall and progression-free survival [7]. The EGFR is a receptor tyrosine kinase that is involved in cell proliferation, survival, differentiation, and migration invasion and metastasis of various cancer types [8,9]. As for OSCC, extensive research in preclinical and clinical settings has shown that EGFR activation leads to the stimulation of proliferative and pro-survival intracellular signaling pathways, such as Mitogen-activated protein kinases (MAPKs) cascade, Phosphoinositide-3 kinase (PI3K)/Akt kinase (AKT)/Mammalian target of rapamycin (mTOR), and the Janus kinase (JAK)/Signal transducer and activator of transcription (STAT) pathway, resulting in increased tumor invasion and metastasis [6,10,11]. All these together suggest that therapeutic targeting of EGFR using anti-EGFR monoclonal antibodies or relevant pathway inhibitors (e.g., tyrosine kinase domain inhibitors) represents a viable option for OSCC patients. However, the efficacy of these therapies may be compromised by pre-existing genetic alterations in the EGFR that confer resistance to EGFR blockades, or the acquisition of secondary mutations that facilitate evasion of targeting under therapeutic pressure. Thus, the discovery of upstream regulatory mechanisms of the EGFR in OSCC may address the existing challenges.

Ras-associated binding (RAB) proteins constitute a diverse family comprising approximately 70 members, each with distinct cellular localization and functions. These RAB proteins play a crucial role in coordinating the biogenesis, transport, and fusion of membrane-bound organelles and vesicles, thereby regulating intracellular membrane trafficking [12]. Previous studies have revealed a correlation between the expression of RAB family proteins and the progression and prognosis of a series of malignancies, including OSCC [13,14]. Importantly, a few findings have unveiled the involvement of RAB27-regulated EGFR in the progression and lymph node metastasis of epithelial tumors such as breast cancer [15]. Researchers have discovered that the activation of FAK and JNK regulates RAB27A-mediated exocytosis, facilitating the secretion of the EGFR, which in turn promotes migration and invasion in breast cancer. Consequently, disrupting the RAB27A-mediated EGFR in breast cancer attenuates the metastatic niche, leading to impaired tumor distant metastasis [15]. However, to the best of our knowledge, there are no reported findings regarding the regulation of abnormally high expression of the EGFR by RAB27 proteins in OSCC.

In this study, we observed a positive correlation between RAB27A expression and the lymph node metastasis in OSCC, which was associated with a worse prognosis. Knockdown of RAB27A resulted in downregulation of proliferation, migration, and invasion of OSCC cell lines in vitro. Further, a transcriptome sequencing analysis revealed that RAB27A regulates migration and invasion through the EGFR signaling pathway. Importantly, we also discovered that RAB27A-mediated palmitoylation, facilitated by the palmitoyl S-acyltransferase ZDHHC13, played a key role in the membrane retention of EGFR, suggesting RAB27A as an upstream regulator of the EGFR in OSCC.

In summary, RAB27A promotes OSCC progression and lymph node metastasis through the regulation of ZDHHC13-mediated palmitoylation in the EGFR signaling pathway. These findings offer valuable insights into the progression of OSCC and underscore the potential of targeting RAB27A as a therapeutic approach to combat this aggressive form of cancer.

## 2. Results

### 2.1. The Overexpression of RAB27A in OSCC

**Figure 1:**
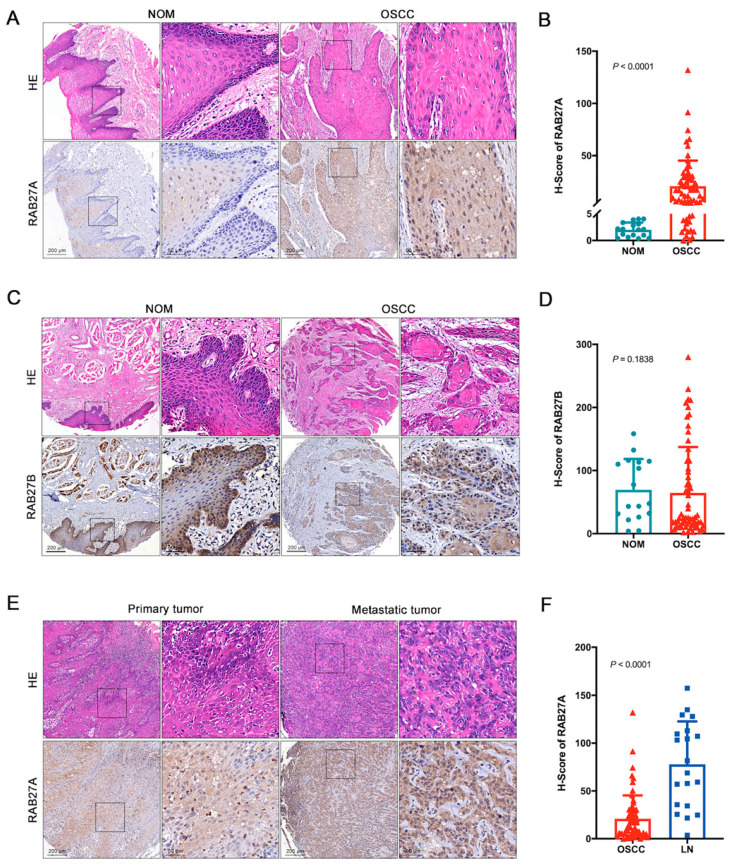
Ras-related protein Rab-27A (RAB27A) is significantly overexpressed in oral squamous cell carcinoma (OSCC) and metastatic lymph nodes. (**A**) Representative images of hematoxylin–eosin (HE) and immunohistochemistry (IHC) staining of RAB27A in normal oral mucosal (NOM) and OSCC tissue. (**B**) Quantification (Dunn’s multiple comparisons test) of the Histoscore (H-Score) of RAB27A among 17 NOM and 63 OSCC, *p* < 0.0001. (**C**) Representative images of HE and IHC staining of RAB27B in NOM and OSCC tissue. (**D**) Quantification of the H-Score of RAB27B among 17 NOM and 63 OSCC, *p* = 0.1838. (**E**) Representative images of HE and IHC staining of RAB27A in primary tumor sites and metastatic tumors in lymph nodes. (**F**) Quantification of the H-Score of RAB27A in primary OSCC tumor sites and paired metastatic tumor sites in lymph nodes, *p* < 0.0001. Scale bar = 200 μm or 50 μm (magnified fields). The magnified view of the tissue images in the black square is shown in the according right panel (**A**,**C**,**E**). Data are presented as means ± SD (**B**,**D**,**F**).

To unmask the expression pattern of the RAB27s in OSCC, we conducted immunohistochemistry staining to analyze the expression levels of RAB27A and Ras-related protein Rab-27B (RAB27B), the two RAB27 isoforms that share high sequence similarity, on tissue microarrays consisting of 17 normal mucosa (NOM) and 63 OSCC samples. The expression level of RAB27A was assessed using the H-score. Our findings revealed a significant upregulation of RAB27A expression in OSCC compared to NOM (Figure 1A,B). As for RAB27B, we observed that its expression level in OSCC was not significantly different from that in NOM (Figure 1C,D). These results suggested that RAB27A, rather than RAB27B, may be involved in the occurrence and progression of OSCC. Considering the potential roles of RAB27A in tumor migration, we subsequently invested the expression level of RAB27A in the metastatic tumor sites within lymph nodes and observed a significant elevation in RAB27A expression compared to the primary sites (Figure 1E,F). These findings indicate that RAB27A may play crucial roles during the dynamic progression period of OSCC and metastasis to lymph nodes.

### 2.2. The Overexpression of RAB27A Is Associated with Enhanced Lymph Node Metastasis and Poor Prognosis of OSCC Patients

**Figure 2:**
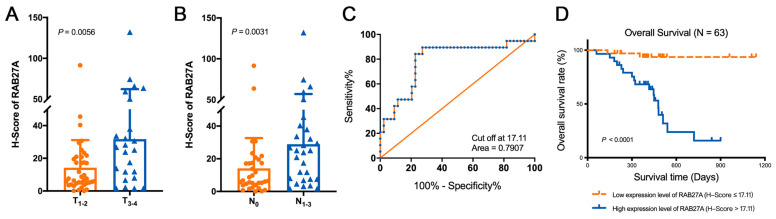
RAB27A significantly correlates with the clinical progression, lymph node metastasis, and survival prognosis of OSCC. (**A**) Comparison of the H-score of RAB27A between OSCC patients with a primary tumor stage (T1–2) and a progressed tumor stage (T3–4), *p* = 0.0056. (**B**) Comparison of the H-score of RAB27A in OSCC patients with or without lymph node metastasis, *p* = 0.0031. (**C**) The receiver operating characteristic (ROC) curve plotted based on the patients’ prognosis, with an area under the curve of 0.7907. (**D**) Comparison of overall survival between RAB27A high or low expression patients using Log-rank (Mantel–Cox) test, *p* < 0.0001. Data are presented as means ± SD (**A**,**B**).

Since high expression of RAB27A is potentially associated with lymph node metastasis in OSCC, we next analyzed the Tumor Node Metastasis (TNM) stages of the patients and found that patients with lower expression levels of RAB27A tended to be in the earlier stage of tumor progression (Figure 2A) and had fewer cases of metastasis to lymph nodes (Figure 2B). A receiver operating characteristic (ROC) analysis identified a cut-off value of 17.11 in the H-score that effectively stratified patients based on survival rate (Figure 2C). Thus, patients were classified into high or low expression levels of RAB27A based on the ROC curve, and those with lower RAB27A expression exhibited significantly better survival outcomes (Figure 2D, *p* < 0.0001). These results strongly suggest that RAB27A plays a crucial role in the occurrence, development, invasion, and lymph nodal metastasis of OSCC and can serve as a predictive indicator for clinical diagnosis and treatment or a potential therapeutic target.

### 2.3. Knockdown of RAB27A Reduces Proliferation and Increases Apoptosis of OSCC Cells

**Figure 3:**
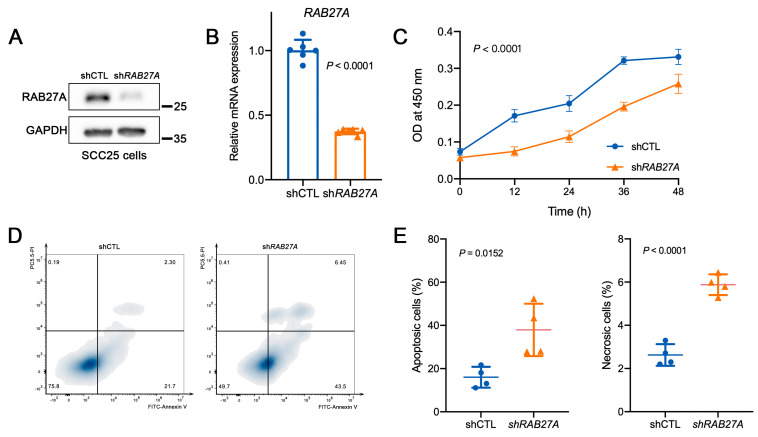
Knockdown of RAB27A decreased proliferation and increased apoptosis in OSCC cells. (**A**) Western blot confirming the knockdown of RAB27A at the protein level. (**B**) Relative mRNA expression of RAB27A in shCTL and sh*RAB27A* cells (*p* < 0.0001). (**C**) Cell proliferation was analyzed by a cell counting kit-8 (CCK8) assay. Growth curve of SCC25 cells with or without knockdown of RAB27A is shown for each treatment at 0, 12, 24, 36, and 48 h (*p* < 0.0001). (**D**) Cell apoptosis was detected by Annexin V-FITC and Propidium (PI) double staining and measured by flow cytometry. (**E**) Percentage of the apoptotic cells (left, *p* = 0.0152) and necrotic cells (right, *p* < 0.0001) in shCTL and sh*RAB27A* cells. Data are presented as means ± SD (**B**,**C**,**E**).

To further elucidate the mechanistic role of RAB27A in the progression of OSCC, we conducted in vitro assays using the OSCC cell line SCC25. The short harpin RNA against human RAB27A (sh*RAB27A*) was packaged into lentivirus and used to transfect the SCC25 cells. Cells transfected with lentivirus carrying empty vectors were used as the control (shCTL). A Western blot analysis was first performed to confirm the successful knockdown of *RAB27A* (Figure 3A), which was simultaneously validated by examining the mRNA expression level of *RAB27A* using real-time quantitative polymerase chain reaction (RT-qPCR) analysis (Figure 3B).

To determine the functions of RAB27A on the biological behaviors of OSCC cells, we next evaluated cell proliferation with a cell counting kit-8 (CCK8) assay, measuring the optical density (OD) values at 450 nm every 12 h. The results revealed a decline in the growth activity of OSCC cells following *RAB27A* knockdown (Figure 3C), suggesting the role of RAB27A in maintaining cell proliferation. Subsequently, we examined apoptosis in sh*RAB27A* cells by detecting Annexin V and Propidium (PI) and analyzing them via flow cytometry (Figure 3D). Annexin V^+^/PI^−^ cells were defined as early apoptotic cells, while Annexin V^+^/PI^+^ cells were classified as late apoptotic or necrotic cells. The findings showed that knockdown of *RAB27A* increased the proportion of apoptotic cells as well as necrotic cells (Figure 3E) in OSCC cells. Taken together, the results suggest that RAB27A sustained the proliferation and prevented the apoptosis of OSCC cells, which might lead to OSCC progression.

### 2.4. Knockdown of RAB27A Attenuates Migration and Invasion Abilities in OSCC Cells

**Figure 4:**
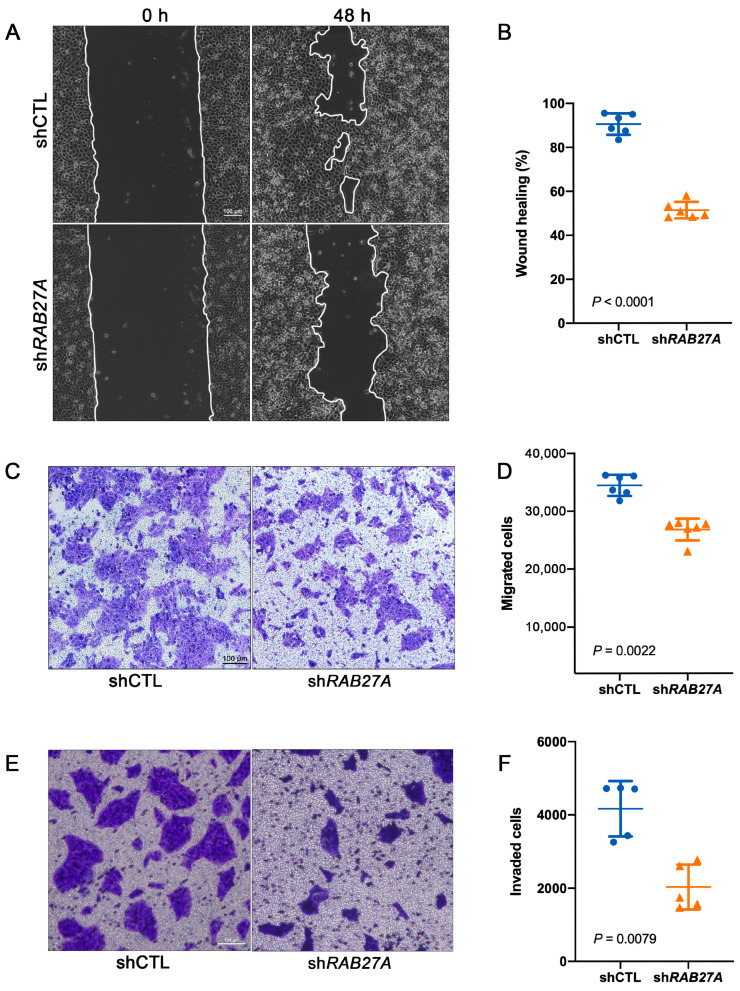
Knockdown of RAB27A impairs the migration and invasion of OSCC cells. (**A**) Representative images of shCTL and sh*RAB27A* cells at 0 h and 48 h post-wounding. Scale bar = 100 μm. (**B**) Quantification of the wound healing percentage of shCTL and sh*RAB27A* at 48 h post-wounding, *p* < 0.0001. (**C**) Representative images of the migrated cells of shCTL and sh*RAB27A* cells. Scale bar = 100 μm. (**D**) Quantification of the number of migrated cells of shCTL and sh*RAB27A*, *p* = 0.0022. (**E**) Representative images of the invaded cells of shCTL and sh*RAB27A* cells. Scale bar = 100 μm. (**F**) Quantification of the number of invaded cells of shCTL and sh*RAB27A*, *p* = 0.0079. Data are presented as means ± SD (**B**,**D**,**F**).

To further study the function of RAB27A in OSCC metastasis, we performed wound healing assays to assess the migration ability of OSCC cells with knockdown of *RAB27A*. Images captured at 0 h and 48 h post-wounding clearly demonstrated a substantial difference in migration between shCTL and sh*RAB27A* cells, as knockdown of *RAB27A* significantly impaired the decrease in wound area when compared to shCTL cells (Figure 4A). Furthermore, the quantification of wound healing percentage corroborated these observations, indicating that RAB27A plays a crucial role in migration capacity of OSCC cells (Figure 4B). We also performed a transwell cell migration assay and revealed that knockdown of *RAB27A* decreased the number of cells detected in the lower chamber (Figure 4C,D), consistent with the results of the wound healing assay.

In order to investigate the invasive ability of OSCC cells, Matrigel was coated on the transwell inserts prior to SCC25 cell seeding. The invasive cells were required to disintegrate the matrix gel and then invade into the lower chamber of the transwell inserts. We found that knockdown of *RAB27A* resulted in almost a 50% reduction in the number of cells that invaded the lower chamber (Figure 4E,F), indicating that RAB27A also plays a crucial role in OSCC invasion.

Taken together, these results suggest that RAB27A is involved in the regulation of OSCC cell migration and invasion.

### 2.5. RAB27A Contributes to the Membrane Retention of EGFR in OSCC Cells

**Figure 5:**
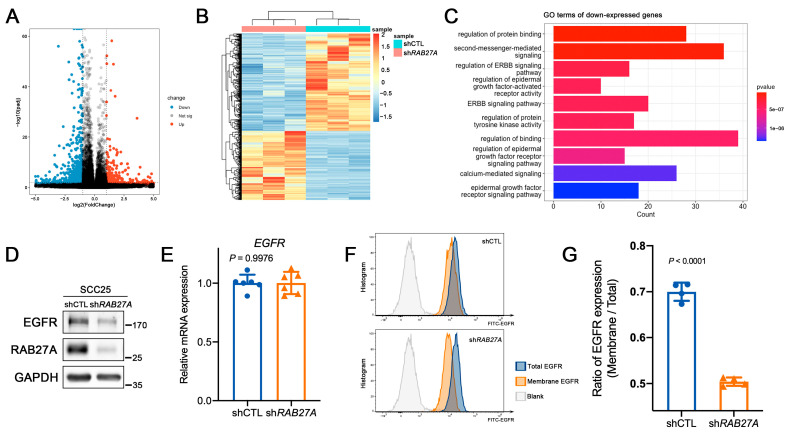
RAB27A contributes to the membrane retention of the EGFR in OSCC cells. (**A**) Volcano plot of differentially expressed genes. Red dots indicate upregulated genes after RAB27A knockdown and blue dots indicate downregulated genes. The *x*-axis represents log2 of fold change and the *y*-axis represents −log10 of adjusted *p* value. (**B**) Heatmap of clustering analysis of shCTL and sh*RAB27A* cells. (**C**) GO enrichment analysis of differentially expressed genes downregulated with RAB27A. The *y*-axis represents the GO terms and the *x*-axis represents the number of target genes. (**D**) Western blot analysis of the expression levels of EGFR in SCC25 cells with *RAB27A* knockdown. (**E**) The relative mRNA expression of EGFR in the shCTL and sh*RAB27A* cells (*p* = 0.9976). (**F**) Representative flow cytometry histogram plots showing the detection of membrane or total EGFR. (**G**) Comparison of the ratio of the membrane EGFR and total EGFR based on the quantitative analysis of the MFI (Mean Fluorescence Intensity), *p* < 0.0001. Data are presented as means ± SD (**E**,**G**).

To investigate the possible mechanism by which RAB27A regulates the biological behaviors of OSCC cells, we extracted the total RNA of shCTL and sh*RAB27A* cells for a RNA sequence analysis. A fold change of ≥2 and an adjusted *p* value of ≤0.05 were considered as statistical significance. A total of 1978 differentially expressed genes between shCTL and sh*RAB27A* cells were identified, including 823 upregulated and 1155 downregulated genes in sh*RAB27A* cells (Figure 5A). A clustering analysis showed significant differences between the two types of cells, providing evidence that RAB27A was intensively involved in the functional regulation of OSCC cells (Figure 5B). Furthermore, a GO functional enrichment analysis was conducted with the differentially expressed genes. Among the top 10 functional terms listed, we discovered that knockdown of *RAB27A* was notably associated with the EGFR signaling pathway (Figure 5C).

To verify this, we performed a Western blot analysis, detecting the expression level of EGFR in sh*RAB27A* SCC25 cells. As expected, knockdown of *RAB27A* significantly decreased the expression level of EGFR in OSCC cells (Figure 5D). Interestingly, the mRNA levels of EGFR did not show any significant reduction after knockdown of *RAB27A* (Figure 5E), suggesting that RAB27A may regulate the EGFR pathway through post-transcriptional modifications. We further examined the distribution of EGFR between the membrane and cytoplasm, and the results demonstrated that silencing *RAB27A* led to a significant decrease in membrane expression of EGFR (Figure 5F,G). This observation suggests that RAB27A might play a role in intracellular trafficking and contribute to membrane retention of the EGFR.

### 2.6. RAB27A-Mediated Palmitoylation of EGFR Sustains the Membrane Stability of the EGFR in OSCC

**Figure 6:**
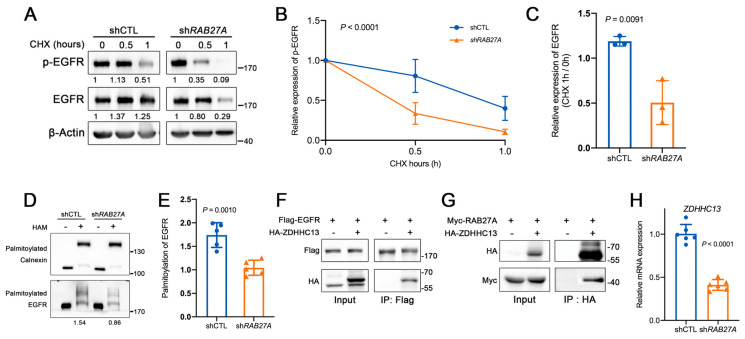
Knockdown of RAB27A disrupts the membrane stability of EGFR via zinc finger DHHC-type containing 13 (ZDHHC13)-mediated palmitoylation. (**A**) Western blot analysis of p-EGFR and EGFR in SCC25 cells pre-treated with 10 ng/mL epidermal growth factor (EGF) for 15 min followed by treatment of 200 ng/mL cycloheximide (CHX). (**B**) The time course of p-EGFR dephosphorylation. p-EGFR expression was normalized to β-Actin grayscale values. The fold change in p-EGFR was determined by comparing CHX-treated cells (0.5 or 1 h) to untreated ones. *p* < 0.0001. (**C**) Ratio of EGFR levels after treatment with CHX for 1 h compared to 0 h in shCTL and sh*RAB27A* cells (*p* = 0.0091). (**D**) APE assay of palmitoylated EGFR in shCTL and sh*RAB27A* cells. Endogenous calnexin, a type I integral membrane protein with two S-palmitoylation sites was detected as a protein loading control for the APE assay. HAM, hydroxylamine. (**E**) Ratio of palmitoylated EGFR to total EGFR expression in the shCTL and sh*RAB27A* cells (*p* = 0.0086). (**F**) The co-immunoprecipitation assay in 293FT cells co-expressing EGFR and ZDHHC13. (**G**) The co-immunoprecipitation assay in 293FT cells co-expressing RAB27A and ZDHHC13. (**H**) The relative mRNA expression of ZDHHC13 in the shCTL and sh*RAB27A* cells (*p* < 0.0001). Data are presented as means ± SD (**B**,**C**,**E**,**H**).

To elucidate the intracellular trafficking of EGFR potentially mediated by RAB27A, we here tested the pace of dephosphorylation of phosphorylated EGFR (p-EGFR) in sh*RAB27A* SCC25 cells stimulated with EGF (10 ng/mL) (Figure 6A). Cycloheximide (CHX) was used to inhibit protein synthesis. We observed that the knockdown of *RAB27A* significantly accelerated the dephosphorylation of p-EGFR (Figure 6A,B) and the degradation of EGFR (Figure 6A,C). These results strongly suggested that RAB27A was pivotal to the maintenance of EGFR activation and stability.

Previous studies also demonstrated that EGFR palmitoylation was essential to its retention to the plasma membrane [16,17]. Therefore, we investigated whether knocking down *RAB27A* affects the levels of EGFR palmitoylation. The acyl-polyethylene glycol exchange (APE) assay results showed that the knockdown of *RAB27A* significantly reduced the palmitoylation level of EGFR (Figure 6D,E). Palmitoylated calnexin here was detected as an internal reference of palmitoylation. Moreover, with a co-immunoprecipitation assay, we revealed that the beads carrying the anti-Flag antibody were able to capture the zinc finger DHHC-type containing 13 (ZDHHC13) protein with HA tag, confirming the interaction between EGFR and ZDHHC13 (Figure 6F), a known catalytic enzyme regulating EGFR palmitoylation and contributing to its membrane retention [18]. To clarify whether RAB27A regulated EGFR palmitoylation through ZDHHC13 and influenced its membrane retention, we co-transfected 293FT cells with both *Myc-RAB27A* and *HA-ZDHHC13*. We detected a direct interaction between RAB27A and ZDHHC13 using co-immunoprecipitation. Moreover, we observed that knockdown of *RAB27A* also led to a decrease in the mRNA expression level of *ZDHHC13* (Figure 6G). These findings collectively indicate that RAB27A can modulate ZDHHC13 at both mRNA and protein levels, thereby affecting its interaction with the EGFR and altering EGFR palmitoylation, which ultimately led to enhanced membrane retention of EGFR in OSCC.

## 3. Discussion

OSCC is a highly aggressive cancer with poor survival rates. While some molecular mechanisms have been identified, challenges persist in improving patient outcomes. Given OSCC’s tendency to migrate, invade, and metastasize, understanding its underlying mechanisms is crucial.

The EGFR plays a significant role in cellular survival processes and has been extensively studied as a biomarker in various cancer types, especially OSCC [19,20]. Notably, up to 80–90% of OSCCs show overexpression or carry mutations in the EGFR, and these alterations significantly affect overall and progression-free survival [7]. Therefore, targeting the EGFR therapeutically with anti-EGFR monoclonal antibodies or kinase inhibitors remains a promising approach for OSCC treatment [21]. However, the effectiveness of these therapies can be compromised by pre-existing genetic alterations or the acquisition of secondary mutations, resulting in therapy resistance [22]. Thus, further research is required to comprehensively characterize the mutational landscape of the EGFR in OSCC. In addition, further understanding the upstream regulatory mechanisms of EGFR aberrations will also enable us to optimize EGFR-targeted therapies, ultimately enhancing the clinical outcomes of OSCC patients. In the present study, we found that the abnormal expression of the EGFR may be closely associated with the elevated expression of RAB27A in OSCC.

RAB27A, like its counterparts in the RAB family, is mainly involved in protein transport and small Guanosine triphosphatase (GTPase)-mediated signal transduction [23,24]. It has been found to be a promoter of tumor progression in many other cancers through regulation of MVB (multivesicular body) trafficking and sEV (small extracellular vesicle) secretion [25,26,27]. Studies have revealed that blockade of RAB27A could therefore decrease the primary tumor growth by suppressing sEV secretion. Interestingly, previous research revealed that RAB27A-mediated exocytosis could be induced by activation of Jun N-terminal kinase (JNK) or focal adhesion kinase (FAK) signaling [15]. This could lead to the secretion of the EGFR, promoting migration and invasion of breast tumor cells. However, the role of RAB27A in OSCC has not been previously reported. Here, in our study, we demonstrated that RAB27A induced a more invasive and metastatic phenotype in OSCC patients, as well as in OSCC cells in vitro. Unlike previous studies focusing on the regulation of tumor sEV secretion led by RAB27A, we found that RAB27A could directly participate in the post-transcriptional regulation of specific proteins within tumor cells. We discovered that RAB27A was closely associated with the EGFR-related signaling pathway in OSCC, according to results from a transcriptome sequencing analysis. However, this association was not simply the regulation of EGFR-carrying small extracellular vesicle (sEV) secretion, but rather the maintenance of EGFR levels on the cell membrane surface. To some extent, this explained the reason for the aberrant expression of the EGFR in OSCC, as the upregulation of RAB27A may act as a potential upstream regulator. Accordingly, knockdown of *RAB27A* in OSCC cells abrogated the membrane retention of the EGFR. However, whether the involvement of sEV secretion of the EGFR could regulate its membrane stability still requires further investigation. Previous research also demonstrated the role of the EGFR and its downstream signaling pathways in sustaining tumor development and homeostasis. Consequently, in the context of this work, the downregulation of RAB27A leads to a reduction in EGFR membrane expression within OSCC cells, thereby diminishing traits driven by the EGFR, such as tumor growth and resistance to apoptosis.

It was noted that palmitoylation of the EGFR played a crucial role in maintaining the stability of membrane-bound EGFR [28]. The DHHC domain is a protein domain that acts as an enzyme, which adds a palmitoyl chemical group to proteins in order to anchor them to cell membranes. It was reported that the EGFR could be palmitoylated by zinc finger DHHC-type containing 20 (ZDHHC20), one of the members of the DHHC family, on the C-terminal [28]. In addition to ZDHHC20, a more recent report highlighted the significance of EGFR palmitoylation by ZDHHC13 in plasma membrane localization, which aligns with the findings of our present study [18]. In this study, we also discovered a direct interaction between ZDHHC13 and RAB27A in OSCC cells, whereas no such interaction was observed with ZDHHC20. This suggests that the regulatory role of RAB27A in the ZDHHC family proteins may not be universal. Meanwhile, knockdown of *RAB27A* resulted in downregulation of *ZDHHC13*, indicating that RAB27A could regulate ZDHHC13 both at the mRNA and protein levels and contribute to mediating EGFR palmitoylation. While we demonstrated the involvement of RAB27A in EGFR palmitoylation and membrane retention, further studies are still needed to dissect the precise molecular mechanisms by which RAB27A regulates ZDHHC13-mediated palmitoylation in the future.

## 4. Materials and Methods

### 4.1. Clinical Samples and OSCC Microarrays

The dysplasia and OSCC samples of microarrays used in this study were collected from the School and Hospital of Stomatology, Wuhan University. Microarrays consisted of 17 samples of normal oral mucosal (NOM), 63 primary OSCC, and 20 metastatic tumors in lymph nodes (LN). All patients provided informed consent, and their prognosis was followed up. The clinical stages and the histological grading of OSCC were classified based on the Union for International Cancer Control (UICC) 2002 guidelines and WHO protocol, respectively.

### 4.2. Immunohistochemistry (IHC) Staining

Tissue samples were fixed with 4% paraformaldehyde, dehydrated, and subsequently paraffin embedded to prepare tissue microarrays. The microarrays were then treated with peroxidase blocker and 5% goat blood serum sequentially, followed by overnight incubation with primary antibodies at 4 °C. Subsequently, the microarrays were treated with species-specific secondary antibodies and avidin-biotin peroxidase. Visualization was achieved using a diaminobenzidine (DAB) kit (MXB Biotechnologies, Beijing, China) and hematoxylin was used for nucleus staining. The microarrays were scanned using an Aperio ScanScope CS scanner (Vista, CA, USA), and the scanned images were quantified with Aperio Quantification software 12.3.3. The Histoscore (H-score) was calculated as follows: H-score = [(percentage of strongly positive staining) × 3 + (percentage of moderately positive staining) × 2 + (percentage of weakly positive staining) × 1)] [29].

### 4.3. Cell Culture

The human OSCC cell line, SCC25 cells, was acquired from the China Center for Type Culture Collection. They were cultured in Dulbecco’s modified Eagle’s medium (DMEM) supplemented with 10% fetal bovine serum (FBS), 100 U/mL penicillin, and 100 mg/mL streptomycin under a humidified atmosphere of 95% air and 5% CO_2_ at 37 °C.

### 4.4. Construction of RAB27A-Knockdown Cells

Lentiviral particles carrying human RAB27A shRNA (Forward sequence 5′→3′: GTTCTTCAGAGATGCTATGC) or shRNA-control (shCTL) were generated using 293FT cells. After 48–72 h, supernatants containing lentiviral particles were harvested. SCC25 cells were then infected with the lentiviral particles and subsequently selected using puromycin (5 μg/mL).

### 4.5. Western Blot Analysis

Using sodium dodecyl sulfate-polyacrylamide gel (SDS-PAGE, 10%), the whole cell lysates of shCTL and sh*RAB27A* SCC25 cells were separated and transferred onto polyvinylidene fluoride (PVDF) membranes (Roche Applied Science, Penzberg, Germany). After that, the blots were incubated with fat-free milk (5%) for 1 h at room temperature. The blots were then incubated with the primary antibodies overnight at 4 °C. Following 1 h incubation with secondary antibodies (HRP-conjugated) at room temperature (RT), the blots were detected by an electrogenerated chemiluminescence (ECL) detection system, and the light emitted was captured as images. For quantification analysis of Western blots, the expression levels of proteins were normalized by the grayscale values of reference proteins.

### 4.6. Cell Proliferation Assay

Briefly, cells (500 cells/well) were seeded in 96-well microplates. Every 12 h, the cells were incubated with the Cell Counting Kit-8 (CCK8) (Biosharp, Hefei, China) for 2 h at 37 °C. The plates were analyzed with a plate reader at a wavelength of 450 nm.

### 4.7. Real-Time Quantitative PCR (RT-qPCR)

Total cell RNAs were isolated and reverse transcribed into cDNA with a PrimeScript 1st Strand cDNA Synthesis Kit (Takara, Otsu, Japan). Additionally, then one-fifth of the cDNA was used for polymerase chain reaction (PCR) with FastStart Universal SYBR Green Master (Roche, Basel, Switzerland) in a RT-qPCR system (Bio-rad, CFX Connect, Hercules, CA, USA). The primer sequences for RT-qPCR are shown in Table 1. The relative quantification in gene expression was determined with 2^−ΔΔCt^ normalization. Briefly, the fold change in the expression of the target gene was initially normalized to an internal control gene (GAPDH) and subsequently further normalized by comparing that of shRAB27A cells to control cells.

### 4.8. Wound Healing Assay

Cells (2 × 10^6^ cells/well) were seeded in 6-well plates. After the cells reached confluency, a scratch was made and then the cells were washed with PBS three times. FBS-free DMEM was used to culture the remaining cells. Images were captured at 0 and 48 h post-wounding. The areas of wound healing were quantified by Image J V1.53.

### 4.9. Cell Migration Assay

Cells (1 × 10^5^ cells/well) were seeded in the upper chamber of transwell inserts with 8 μm pores (Jet Bio-Filtration Co., Guangzhou, China) with FBS-free DMEM, while DMEM with 10% FBS was added to the lower chamber. After incubation at 37 °C for 24 h, the cells in the lower chamber were fixed with 4% paraformaldehyde for 15 min and stained with 0.1% crystal violet for 20 min at room temperature and the upper cells were removed with cotton swabs. Images were captured, and the number of invaded cells was quantified by Image J V1.53.

### 4.10. Cell Invasion Assay

Transwell inserts with 8 μm pores (Jet Bio-Filtration Co., Guangzhou, China) were coated with 20 μg of matrix gel (Corning, Corning, NY, USA) and incubated at 37 °C for 30 min. Cells (5 × 10^5^ cells/well) were then seeded in the upper chamber with FBS-free DMEM, while DMEM with 10% FBS was added to the lower chamber. After incubation at 37 °C for 48 h, the cells in the lower chamber were fixed with 4% paraformaldehyde for 15 min and stained with 0.1% crystal violet for 20 min at room temperature. The upper cells were removed with cotton swabs, and images were captured. The number of invaded cells was quantified using Image J V1.53.

### 4.11. Flow Cytometry

shCTL and sh*RAB27A* cells were cultured and harvested during the logarithmic growth phase and washed twice with PBS. The cells were permeabilized and fixed before detecting the plasma proteins, while for the membrane proteins, cells were not permeabilized. Cells were incubated with specific antibodies for 30 min at 4 °C and then washed and resuspended in PBS for detection with a flow cytometer. The flow cytometry data were analyzed using FlowJo software 10.8.1 to generate histograms and quantify the MFI (Mean Fluorescence Intensity).

### 4.12. Bioinformatic Analysis

For bioinformatic analyses, R software (V.4.2.0) and relevant R packages were utilized. DESeq2 was employed to calculate the differentially expressed genes, and a Gene Ontology (GO) enrichment analysis was conducted to screen the molecular mechanisms involved. A significance level of an adjusted *p* value (padj) of *<*0.05 was considered statistically significant.

### 4.13. Detection of Palmitoylation of Protein

Palmitoylated proteins were detected using the Acyl-PEG exchange (APE) assay as previously described [30]. Briefly, cells were lysed in TEA lysis buffer with 5 mM EDTA, 4% SDS, and protease inhibitors and sonicated at room temperature. The cell lysate was treated with Tris (2-carboxyethyl) phosphine (TCEP, #20490, Pierce, Appleton, WI, USA) for 30 min at room temperature with nutation, and then with N-ethylmaleimide (NEM, #E1271, Sigma, St. Louis, MO, USA) for 2 h to reduce and block the free cysteine residues. The proteins were precipitated using a methanol–chloroform–deionized H_2_O procedure (prechilled, 8:3:6). TEA lysis buffer with 5 mM EDTA and 4% SDS was used to dissolve the protein, followed by treatment with 1 M hydroxylamine (HAM, #379921, Sigma, St. Louis, MO, USA) for 1 h at room temperature with nutation to remove the S-fatty acid groups. Another methanol–chloroform–deionized H_2_O procedure was performed, and then the exposed cysteines were incubated with 1.33 mM 10 kDa mPEG-Mal (#712469, Sigma-Aldrich, St. Louis, MO, USA) for 2 h at room temperature with nutation. The proteins were isolated by the methanol–chloroform–deionized H_2_O procedure and boiled in SDS loading buffer at 95 °C for 5 min. The palmitoylated proteins were analyzed by Western blotting and Image J.

### 4.14. Co-Immunoprecipitation

HEK293 cells transiently transfected with the target protein were lysed using IP-RIPA buffer on ice. The sample lysates were then incubated with beads that bind specific antibodies against the target protein for 4 h while gently agitating to promote binding between the beads and the antibody–antigen complexes. After thoroughly washing with buffer to remove any non-specifically bound proteins. The proteins on the beads were boiled in SDS loading buffer at 95 °C for 5 min and analyzed by Western blotting.

### 4.15. Statistical Analysis

For statistical comparisons between NOM and OSCC, as well as the association between RAB27A and clinicopathologic characteristics of OSCC, Student’s *t* tests were employed. The Log-rank (Mantel–Cox) test was used to analyze the survival rate of OSCC patients. A significance level of *p* < 0.05 was considered statistically significant. Statistical analyses were performed using GraphPad Prism 9.0.

## 5. Conclusions

Our study provided novel insights into the role of RAB27A in OSCC, demonstrating that RAB27A regulates ZDHHC13-mediated palmitoylation of EGFR, resulting in enhanced migration, invasion, and lymph nodal metastasis of OSCC. It also underscored the potential of targeting RAB27A as a therapeutic approach to improve the clinical outcomes for OSCC patients.

## Figures and Tables

**Table 1:** Primer sequences for RT-PCR.

Gene	Forward 5′→3′	Reverse 5′→3′
*GAPDH*	GGAGCGAGATCCCTCCAAAAT	GGCTGTTGTCATACTTCTCATGG
*RAB27A*	GCTTTGGGAGACTCTGGTGTA	TCAATGCCCACTGTTGTGATAAA
*EGFR*	AGGCACGAGTAACAAGCTCAC	ATGAGGACATAACCAGCCACC
*ZDHHC13*	ACCCCACTCTTATTGATGGAGA	TGTCTGCCCATTTACATCTGTC

## Data Availability

The data that support the findings of this study are available from the corresponding author upon reasonable request.
